# Diet-induced dampness-heat psoriasis is characterized by reduced *Lactobacillus* and accumulation of deoxycholic acid

**DOI:** 10.3389/fcimb.2026.1704547

**Published:** 2026-03-02

**Authors:** Yihan Wang, Fengling Xing, Qiujun Zhou, Hui Li, Hongyang Fu, Fan Zhang, Xiaohong Yang, Yi Cao

**Affiliations:** 1 The Second Affiliated Hospital, Zhejiang Chinese Medical University, Hangzhou, China; 2 Dermatology Department, Hangzhou TCM Hospital Affiliated to Zhejiang Chinese Medical University, Hangzhou, China; 3 The First School of Clinical Medicine of Zhejiang Chinese Medical University, Hangzhou, China; 4 Department of Dermatology, The First Affiliated Hospital of Zhejiang Chinese Medical University (Zhejiang Provincial Hospital of Chinese Medicine), Hangzhou, China; 5 Department of Radiology, The First Affiliated Hospital of Zhejiang Chinese Medical University (Zhejiang Provincial Hospital of Chinese Medicine), Hangzhou, China

**Keywords:** bile acid metabolism, deoxycholic acid, endogenous dampness-heat syndrome, gut microbiota, *Lactobacillus*, psoriasis

## Abstract

**Background:**

Psoriasis is a common immune-mediated skin disease influenced by environmental and dietary factors. In traditional Chinese medicine (TCM), endogenous dampness-heat syndrome, often induced by diets rich in stimulating foods, is considered a trigger that aggravates psoriasis. However, the underlying mechanisms remain unclear. This study investigated the gut microbiota and metabolic alterations associated with endogenous dampness-heat syndrome in psoriasis.

**Materials and methods:**

BALB/c mice were fed a stimulating food diet to establish a model of endogenous dampness-heat syndrome, followed by the induction of psoriasis-like dermatitis by applying imiquimod. Mice on a standard diet served as disease controls and healthy controls. Characteristics of the gut microbiota were analyzed by 16S rDNA sequencing. UPLC–MS/MS was used to detect metabolic changes in the feces and serum of mice and to quantify multiple bile acids. Lipid accumulation and bile acid content in the liver were evaluated by Oil Red O staining and total bile acid assays.

**Results:**

Endogenous dampness-heat modeling aggravated psoriasis-like symptoms in mice. This was accompanied by marked dysbiosis of the gut microbiota, characterized by reduced abundance of *Lactobacillus* and *Bacteroides*. Serum and fecal metabolomics revealed prominent alterations in bile acid metabolism, closely associated with the reduction in *Lactobacillus*. Targeted quantification confirmed elevated deoxycholic acid in serum, together with increased total bile acids and lipid deposition in the liver. The expression of FXR in bile acid pathway in the liver was decreased, while the expression of CYP7A1 was increased.

**Conclusion:**

The exacerbation of skin lesions and hepatic lipid deposition in endogenous dampness-heat pattern psoriasis may be associated with bile acid imbalance and reduced Lactobacillus levels.

## 1 Introduction

Psoriasis is an immune-mediated inflammatory skin disease that can occur at any age (Boehncke and Schön, 2015).Psoriasis is clearly associated with gastrointestinal and metabolic diseases, suggesting systemic involvement beyond the skin (Takeshita et al., 2017; Gisondi et al., 2018; Griffiths et al., 2021). In traditional Chinese medicine (TCM), an improper diet is thought to impair spleen and stomach function, leading to endogenous dampness-heat (Yang et al., 2014). This is also the most common sydrome of psoriasis. Clinically, the skin lesions in these patients are characterized by a bright red and moist appearance, with yellowish, greasy scales, and are accompanied by symptoms such as obesity, fatigue, abdominal distension and loose stools (Societies WFoCM, 2024). Such dietary patterns, often referred to as “stimulating foods, “ are recognized as important triggers that aggravate psoriatic symptoms (Zeng et al., 2023). Characterized by high fat and sugar, as well as pungency, they are believed to impair the spleen and stomach’s function, while promoting dampness and generating heat (Yao et al., 2017; Spleen and Stomach Diseases Branch CAoCM, 2024). Clinical evidence supports the role of endogenous dampness-heat in worsening psoriasis (Zhang et al., 2022), but more mechanistic studies are still needed.

Increasing evidence suggests that many of TCM-defined syndromes correspond to identifiable metabolic or microbiota-related abnormalities. Diet-induced dampness-heat is closely associated with gut microbiota dysbiosis (Qiao et al., 2022; Chen et al., 2024). Given the recognized bidirectional communication along the gut–skin axis, disturbances in gut microbiota and its metabolites have been increasingly implicated in the pathogenesis of psoriasis (Olejniczak-Staruch et al., 2021; De Francesco and Caruso, 2022; Mahmud et al., 2022; Sonomoto et al., 2023; Chen et al., 2024). The abundance of *Bifidobacterium* and *Lactobacillus* in the patient’s guts decreased, while the abundance of pathogenic taxa such as *Salmonella* and *Campylobacter* increased (Myers et al., 2019). These alterations suggest that the gut microbiota may contribute to disease processes (Shi et al., 2021; Thye et al., 2022). Additionally, the gut microbiota is directly influenced by food and serve as a core mechanism for dietary intervention in psoriasis (Yu et al., 2019; Beam et al., 2021; García-Montero et al., 2021; Malesza et al., 2021; Sonomoto et al., 2023). *Anaerostipes hadrus* is enriched in psoriasis patients consuming a red-meat diet, mediating the relationship between dietary intake and disease severity (Xiao et al., 2025). High-fat and high-sugar diets can aggravate psoriasis by increasing *Ruminococcaceae* and *Lachnospiraceae* (Shi et al., 2021; Constantin et al., 2025). Based on this, we consider that diet-induced endogenous dampness-heat syndrome also depends on gut microbiota–mediated effects on psoriasis.

In previous studies, we established a mouse model of endogenous dampness-heat syndrome of psoriasis and demonstrated that enhanced M1 macrophage polarization in the intestine contributes to disease progression (Zeng et al., 2023). Building on this model, this study aimed to comprehensively characterize the gut microbiota and metabolic alterations in dampness-heat psoriasis. We hypothesized that diet-induced dampness-heat exacerbates psoriasis through disruption of the gut microbiota and metabolism.

## 2 Materials and methods

### 2.1 Reagents

Imiquimod (IMQ) cream was purchased from Inova Pharmaceuticals Pty, Ltd. (Singapore). The E.Z.N.A.^®^ stool DNA kit was purchased from Guangzhou Feiyang Biotechnology Co. Ltd. (Guangzhou, China). Methanol was purchased from TEDIA (USA). Red oil O powder (O8010) was purchased from SolarBio (Beijing, China).

The modeling feed was prepared according to our previous protocol (Zeng et al., 2023). Egg yolk powder (S30910), sucrose (S11055), and soybean oil (S24362) were purchased from Shanghai Yuan Ye Biotechnology Co., Ltd. (Shanghai, China); fennel, ginger, angelica, okra, small cardamom, and pepper were purchased from the outpatient department of Zhejiang University of Traditional Chinese Medicine (Hangzhou, China). Mouse maintenance feed (1010088) was purchased from Xietong Biology Co. Ltd. (Nanjing, Jiangsu, China). In brief, these herbs were boiled, soaked in soybean oil, and then incorporated into the maintenance feed together with egg yolk powder, sucrose and other components. The formulations for the maintenance feed and the modeling feed are presented in **Tables 1**, **2**. Calculation of energy content and percentage of Recommended Daily Allowance (%RDA) for the maintenance diet have been calculated and included in **Table 1**. All detailed calculation procedures and reference standards are now provided in **Supplementary Table 1**.

**Table 1:** Normal dietary ingredient composition (% by weight) of the maintenance feed.

Ingredient	g/kg diet	kcal/kg	% RDA
Casein, 30 Mesh	200	800	143
L-Cystine	3	12	100
Corn starch	397	1588	100
Maltodextrin 10	132	528	100
Sucrose	100	400	100
Cellulose	50	0	N/A
Soybean oil (no additives)	70	630	100
t-butylhydroquinone	0.014	0	N/A
Mineral Mix S10022M	35	0	100
Vitamin Mix V10037	10	0	100
Choline Bitartrate	2.5	0	100
Total	1, 000	3958	/

**Table 2:** Proportion of constituents of modeling feed.

Supplement regimen	Proportion
Normal diet of mice	60%
Soybean Oil (with additives^1^)	10%
Yolk	15%
Butter	5%
Dried milk powder	7%
Sugar	3%

^1^Crushed plant spices (fennel 3 g, Chinese ginger 5 g, radix angelicae 4 g, Amomum tsao-ko 4 g, cardamon 5 g and pepper 3 g) in 20% volume dose of oil, simmered for 20 min.

### 2.2 Establishment of animal model and sample collection

Eighteen 6-week-old male specific pathogen-free (SPF) BALB/c mice were purchased from the Animal Experiment Center of Zhejiang University of Traditional Chinese Medicine (Hangzhou, China). The mice were reared in an SPF barrier facility under controlled conditions (temperature: 20–24 °C, humidity: 55 ± 10%, and a 12-hour light/dark cycle). All animal procedures were approved by the Animal Experiment Center of Zhejiang University of Traditional Chinese Medicine (Approval No.: IACUC-20201109-07).

After one week of acclimatization, the mice were randomly divided into three groups: control (CON), psoriasis (PSO), and stimulating food (SF) (n = 6). The CON and PSO groups received standard maintenance feed, whereas the SF group was fed the modeling feed. All mice had free access to food. In week 11, the dorsal skin (approximately 2.5 × 2.5 cm²) of each mouse was shaved. IMQ cream (62.5 mg, 5%) was applied topically once daily for 7 days in the PSO and SF groups, whereas Vaseline was applied in the CON group. In summary, the PSO group received IMQ intervention alone, while the SF group received both the modeling diet and IMQ intervention. The model of endogenous dampness-heat syndrome was identified based on characteristic manifestations (**Table 3**). Skin lesions were assessed by gross observation and dermatoscopy. The Psoriasis Area and Severity Index (PASI) score, including erythema, scaling, and infiltration (0–4 each), and skin thickness were recorded.

**Table 3:** Clinical symptoms of dampness-heat syndrome and corresponding mouse manifestations.

Clinical symptom	Mouse manifestation
heavy cumbersome limbs	Reduced sensitivity, sluggish response
Abdominal fullness and distention, nausea and retching	Reduced food intake
Thirst without large fluid intake	Reduced water intake
Sloppy diarrhea, burning sensation in the anus	Anal redness and swelling, loose stools around the anus
Yellow urine	Yellow urine

After modeling, cecal feces were collected. Blood samples were collected, allowed to clot for 1 hour, centrifuged to obtain serum, and stored at −80 °C. Skin and liver of the mice were collected and stored in 4% paraformaldehyde. The spleen was weighed to calculate the spleen index (spleen index = spleen weight (mg)/[body weight (g) × 10]).

### 2.3 Histology (hematoxylin and eosin staining)

Skin tissues were dehydrated in graded concentrations of ethanol, cleared in xylene, and embedded in paraffin. Sections (5 μm) were deparaffinized, rehydrated, stained with hematoxylin, differentiated with acid alcohol, blued, counterstained with eosin, and mounted in neutral resin. Pathological changes were examined under a light microscope. Histopathological alterations associated with psoriasis were analyzed using Baker’s scoring system (Luo et al., 2016).

The liver was dehydrated in a graded sucrose solution and embedded in optimal cutting temperature (OCT) compound. After sectioning and staining, the same staining method as for the skin sections was used, and structural changes in the liver tissue were observed after sealing. Nonalcoholic fatty liver disease Activity Score (NAS) is used to evaluate hepatic steatosis.

### 2.4 Oil red O staining

Frozen liver sections were equilibrated to room temperature, rinsed, and stained with freshly prepared Oil Red O working solution after pretreatment with 60% isopropanol. Sections were subsequently rinsed with isopropanol, counterstained with hematoxylin, differentiated, blued, and mounted. Lipid droplets were visualized under a microscope.

### 2.5 16S rDNA sequencing

Total genomic DNA of the intestinal microbiota was extracted from feces using a DNA extraction kit, and the quality of DNA extraction was checked by agarose gel electrophoresis. DNA concentration was quantified using a UV spectrophotometer. The V3–V4, V4, and V4–V5 hypervariable regions were amplified and sequenced on an Illumina MiSeq platform. After purification, the amplicons were evenly combined and sequenced, according to the manufacturer’s instructions. Qualified libraries were analyzed using an Agilent 2100 Bioanalyzer (Agilent, USA) and sequenced on Illumina platforms (Kapa Biosciences, Woburn, MA, USA). Sorting was performed using the NovaSeq PE250 platform. Paired-end sequences were clustered into operational taxonomic units (OTUs). Demultiplexing was performed using DADA2 to obtain feature tables and sequences, with each OTU’s representative sequence assigned a classification.

### 2.6 Serum and fecal metabolomics

Metabolites were extracted using 120 μL of precooled 50% methanol, vortexed for 1 min, and incubated at room temperature for 10 min. After centrifuging at 4000g for 20 min, the supernatant was transferred to a new 96-well plate. Chromatographic separation was performed using an ultra-high-performance liquid chromatography (UPLC) system (SCIEX, UK) equipped with an ACQUITY UPLC T3 column (100 mm*2.1 mm, 1.8 μm, Waters, UK). A high-resolution TripleTOF 5600 plus tandem mass spectrometer (SCIEX, UK) was used for mass spectrometric analysis in both the positive and negative ion modes. The original data files were converted to mzXML format and processed using the XCMS, CAMERA, and metaX toolboxes included in the R software. The open-source databases KEGG and HMDB were used for metabolite annotation. Bioinformatic analysis was performed using the OmicStudio tools at https://www.omicstudio.cn/tool.

### 2.7 Bile acid quantification

Samples were diluted with water and extracted with a methanol-acetonitrile mixture containing internal standards. Dried extracts were reconstituted in acetonitrile–water, centrifuged, and supernatants collected. Chromatographic separation was performed on a Waters Atlantis Premier BEH ZHILIC column (1.7 μm, 2.1 × 150 mm) using a Waters ACQUITY UPLC H-Class system. Mass spectrometric detection was carried out on a SCIEX 6500 QTRAP with data processed using SCIEX Analyst WorkStation Software (v1.7.2).

### 2.8 Enzyme-linked immunosorbent assay

The levels of TNF-α, IL-6 and IL-17A in serum were measured by ELISA. Reagents were purchased from Jingmei Biotech (China). The content of the above inflammatory factors was detected according to the instruction.

### 2.9 Triglyceride assay

Liver homogenates were prepared using anhydrous ethanol. Liver triglyceride content was measured using a quantitative triglyceride assay kit (Nanjing Jiancheng Bioengineering Institute, China). Optical density (OD) was read at 500 nm and used for subsequent calculations.

### 2.10 PCR

Total RNA was extracted using TRIzol reagent. Following reverse transcription, the expression of FXR, TGR5, and CYP7A1 in liver tissue was detected using 2x Super SYBR Green qPCR Master Mix (Yishan Biotechnology Co., Ltd, China). The primer sequences were as follows: FXR: F: 5’ -CCCCTGCTTGATGTGCTAC-3’, R: 5’-CGTGGTGATGGTTGAATGTC-3’. CYP7A1: F:5’-CTGGGCTGTGCTCTGAAGT-3’, R:5’-GGGAGTTTGTGATGAAGTGGA-3’. TGR5: F: 5’- CCTGGCAAGCCTCATCGTC-3’, 5’- AGCAGCCCGGCTAGTAGTAG-3’. Actin: F: 5’-CACTCTTCCAGCCTTCCTTC-3’, R: 5’-GTACAGGTCTTTGCGGATGT-3’.

### 2.11 Data analysis

Statistical analyses were performed using SPSS version 23.0 (IBM, Chicago, IL, USA). For normally distributed data with equal variances, analysis of variance (ANOVA) was performed, followed by independent sample t-tests for pairwise comparisons. Non-parametric tests were used for data with unequal variances. Correlations were analyzed using Spearman’s rank correlation. Graphs were generated in GraphPad Prism 8.0 (GraphPad Software, La Jolla, CA, USA). Data are presented as mean ± SD, and *p<0.05* was considered statistically significant.

## 3 Results

### 3.1 Endogenous dampness-heat syndrome exacerbates psoriasis symptoms in mice

**Figure 1:**
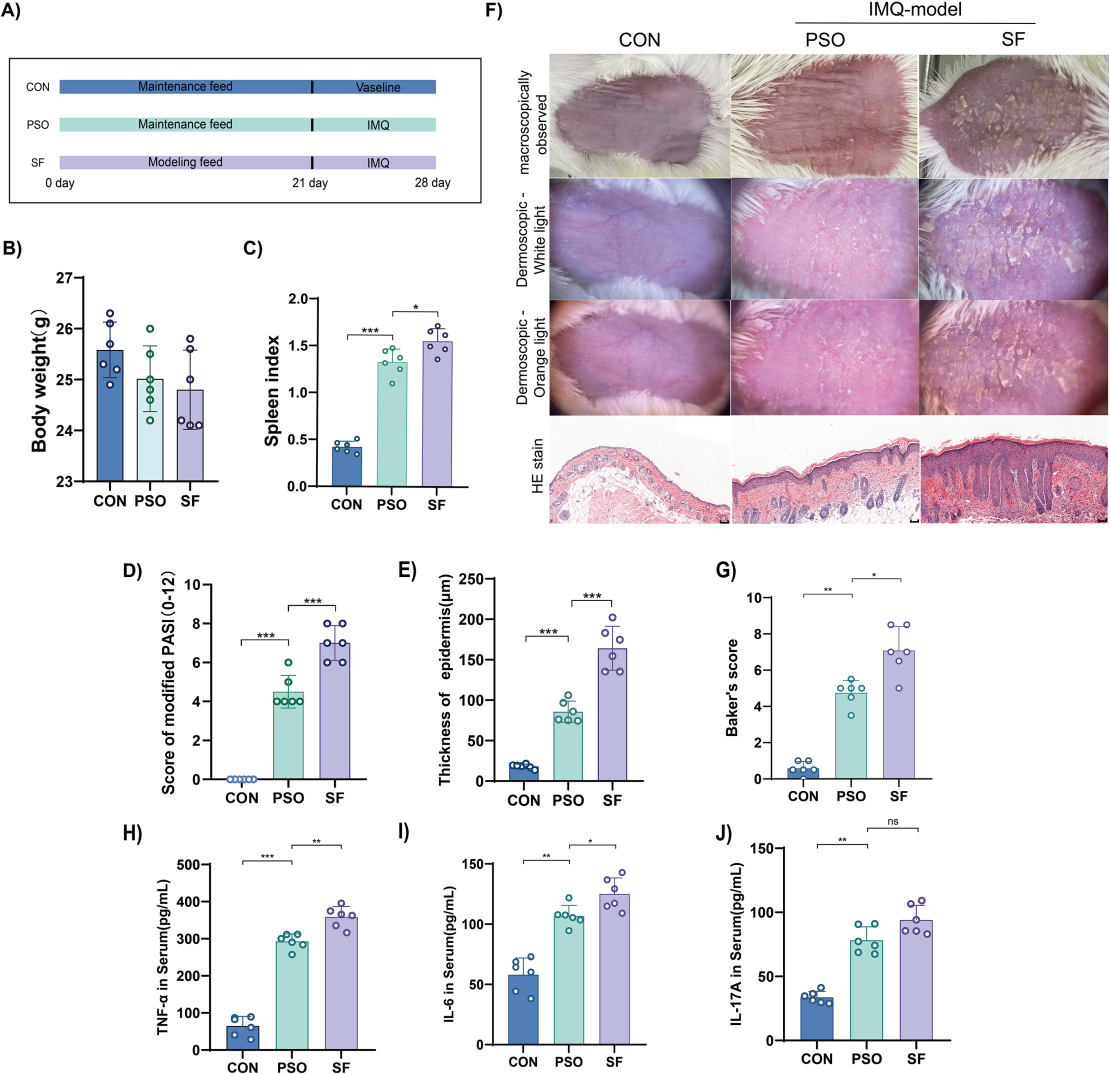
Endogenous dampness-heat syndrome exacerbates psoriasis symptoms in mice (n=6). **(A)** Experimental workflow. **(B)** Body weight. **(C)** Spleen index. **(D)** PASI score. **(E)** Skin thickness. **(F)** Representative skin lesions, dermoscopic images (parallel- and cross-polarized light), and H&E-stained sections. **(G)** Baker’s score. **(H)** Level of TNF-α in serum. **(I)** Level of IL-6 in serum. **(J)** Level of IL-17A in serum. Scale bar=50μm. *p<0.05, **p<0.01, ***p<0.001.

Mice in the SF group were first fed the modeling diet for 21 days to induce dampness-heat syndrome. Subsequently, PSO and SF mice received topical IMQ application on their backs for 7 days (**Figure 1A**). Mice in the SF group showed symptoms of endogenous dampness-heat syndrome, such as anal redness and swelling, and loose stools. Differences in skin lesions were recorded through direct observation and dermatoscopy. Skin thickness, PASI score, and histopathology were used to evaluate skin lesions. Consistent with our previous findings, body weight did not differ significantly among groups (**Figure 1B**). Compared with the PSO group, the SF group showed an increased spleen index (**Figure 1C**), as well as significantly higher PASI scores and greater epidermal thickness (**Figures 1D, E**). Dermatoscopy revealed more severe scaling and epidermal thickening in SF mice under parallel-polarized light, while cross-polarized light demonstrated more pronounced erythema. Histological analysis confirmed aggravated hyperkeratosis and lymphocyte infiltration in the SF group (**Figure 1F**). Baker’s score indicated that the SF group exhibited more severe psoriatic pathology than the PSO group (**Figure 1G**). Serum inflammatory cytokine analysis revealed significantly higher levels of TNF-α and IL-6 in the SF group compared with the PSO group, while IL-17A levels were marginally higher but not statistically significant (**Figures 1H–J**). The results indicate that endogenous dampness-heat syndrome aggravates psoriasis-like manifestations.

### 3.2 Endogenous dampness-heat syndrome aggravates gut microbiota dysbiosis in psoriasis

**Figure 2:**
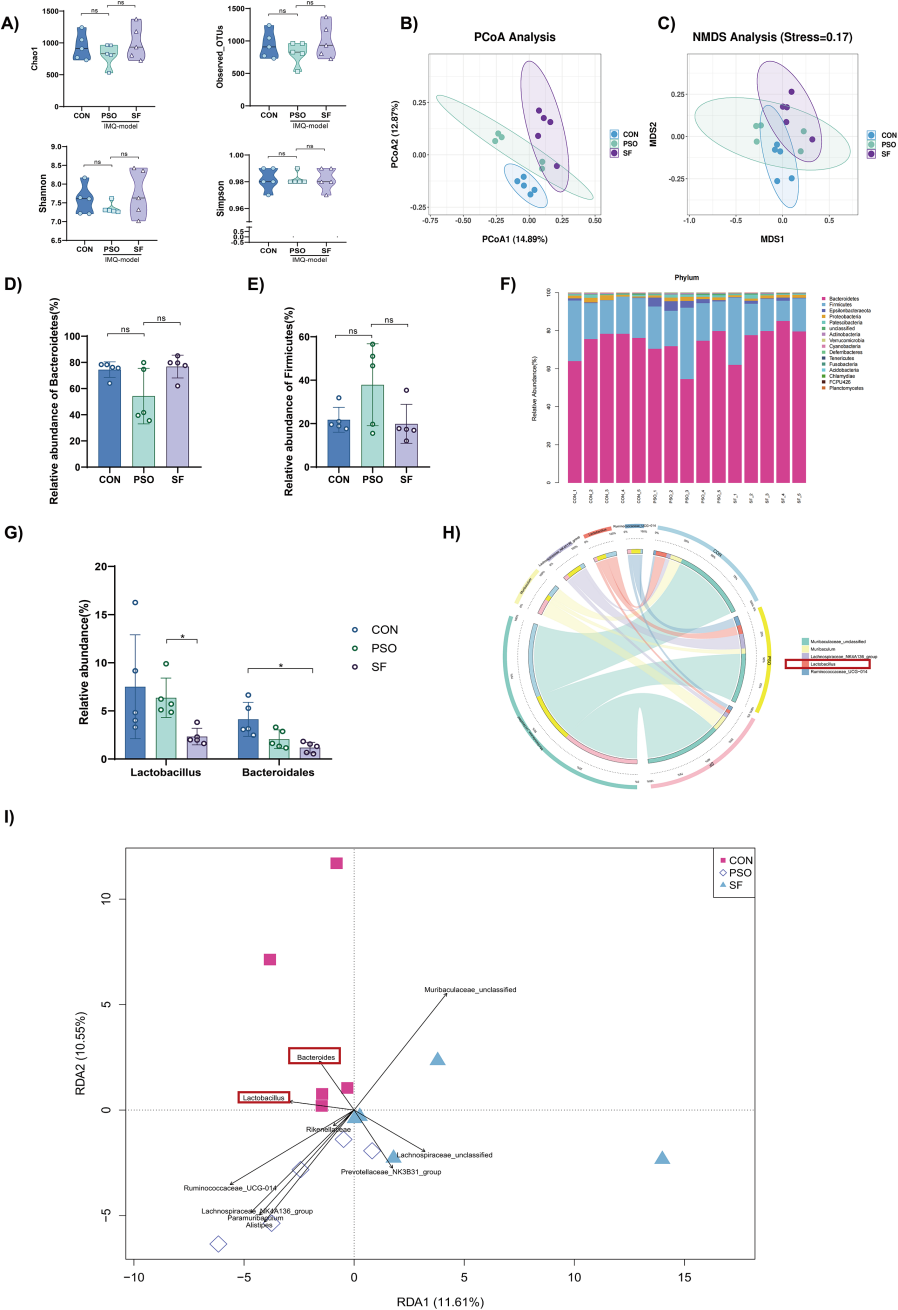
Characteristics of gut microbiota in the three groups (n=5). **(A)** Alpha diversity indices. **(B, C)** PCoA and NMDS plots. **(D, E)** Relative abundance of *Firmicutes* and *Bacteroidetes.* **(F)** Gut microbiota composition in the phylum. **(G)** Relative abundance of *Bacteroides* and *Lactobacillus*. **(H)** Circos plot of the top 5 genera with differential abundance. **(I)** RDA analysis at the genus level. *p<0.05.

To investigate the gut microbiota characteristics of endogenous dampness-heat psoriasis, we used 16S rDNA sequencing. Alpha diversity indices (Chao1, Observed OTUs, Shannon, Simpson) did not differ significantly among groups (*p > 0.05*) (**Figure 2A**). However, beta diversity analysis using PCoA and NMDS revealed distinct clustering, with the SF group showing the greatest separation from the controls (**Figures 2B, C**).

At the phylum level, the relative abundances of *Firmicutes* and *Bacteroidetes* did not differ significantly (**Figures 2D–F**). Significant alterations were observed at the genus level. Both PSO and SF groups displayed reduced abundances of *Bacteroides* and *Lactobacillus*, with the lowest levels in SF mice (*p < 0.05*) (**Figure 2G**). These taxa contributed substantially to overall genus-level differences, as shown by Circos and redundancy analysis (**Figures 2H, I**). Collectively, these results suggest that endogenous dampness-heat syndrome exacerbates gut microbiota dysbiosis at the genus level.

### 3.3 Fecal metabolic characteristics of psoriasis mice with endogenous dampness-heat syndrome

**Figure 3:**
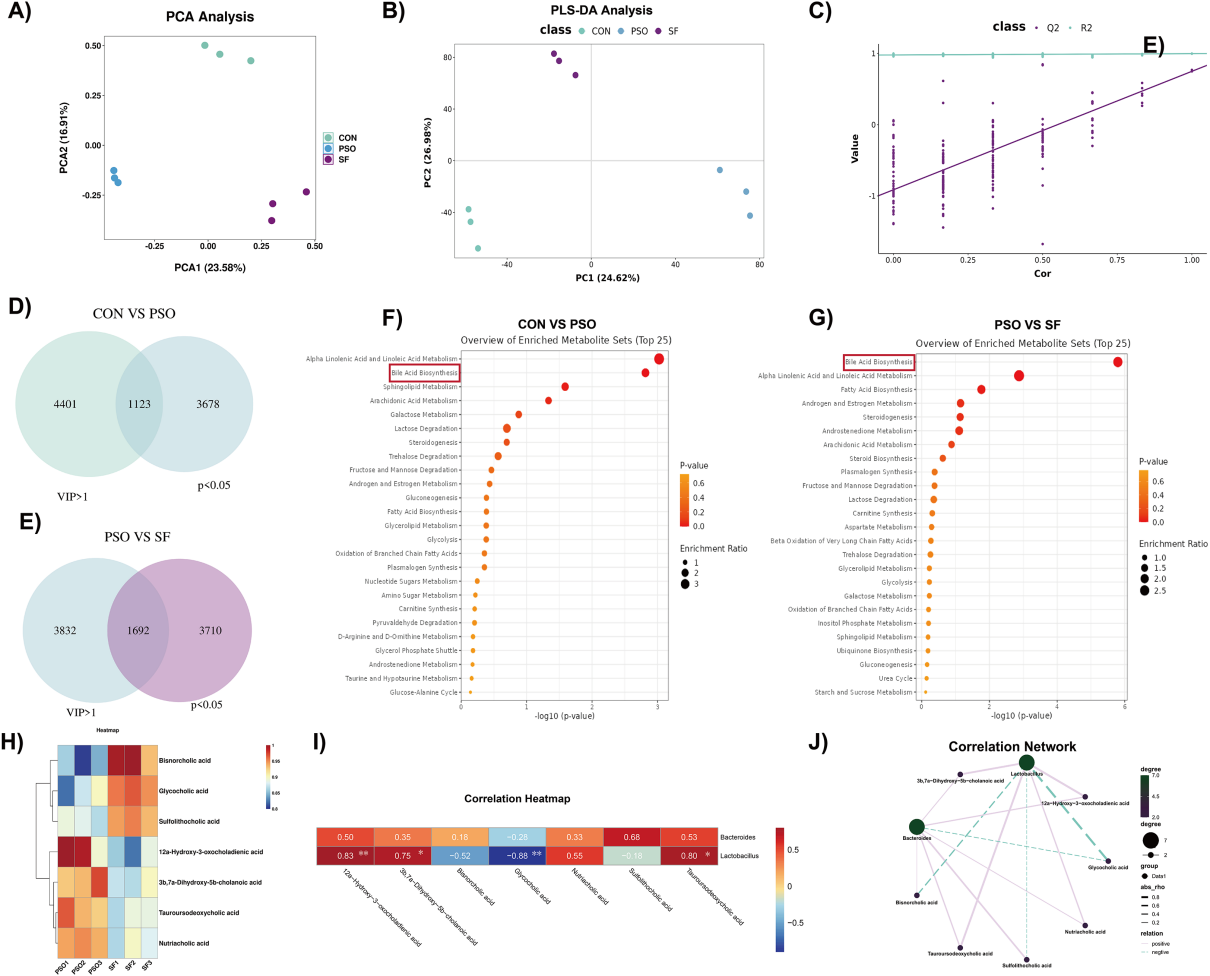
Differences of fecal metabolites among the three groups (n=3). **(A)** Principal component analysis (PCA) plot shows the overall distribution and clustering of fecal metabolite profiles among the CON, PSO and SF groups. **(B)** Partial least squares discriminant analysis (PLS-DA) score plot illustrates group separation based on fecal metabolomic profiles. **(C)** Permutation test of the PLS-DA model evaluates model reliability. **(D)** Using variable importance in projection (VIP) > 1 and *p < 0.05* as selection criteria, the Venn diagram illustrates the number of differentially abundant fecal metabolites between the CON (green) and PSO (blue) groups. **(E)** Using VIP > 1 and *p < 0.05* as selection criteria, the Venn diagram illustrates the number of differentially abundant fecal metabolites between the PSO (blue) and SF (purple). **(F)** Pathway enrichment plot of differentially fecal metabolites between the CON and PSO groups based on the KEGG database. **(G)** Pathway enrichment plot of differentially fecal metabolites between the PSO and SF groups based on the KEGG database. **(H)** Heat maps showing the difference in bile acids between the PSO group and the SF group. **(I, J)** Correlation heat maps and correlation network maps showing the correlation between microbiota and different bile acids. *p<0.05, **p<0.01.

Fecal metabolites were profiled using UPLC-TripleTOF-MS. Principal Component Analysis (PCA) and Partial Least Squares Discriminant Analysis (PLS-DA) indicated distinct fecal metabolic characteristics among the groups (**Figures 3A, B**), and PLS-DA permutation test evaluating model reliability and overfitting (**Figure 3C**). Differential fecal metabolites between the CON and PSO groups and the PSO and SF groups were determined using univariate (*p < 0.05*) and multivariate statistics (VIP>1). Differential metabolite analysis identified 1123 altered metabolites between the CON and PSO groups and 1692 between the PSO and SF groups (**Figures 3D, E**). Pathway enrichment revealed significant alterations in bile acid metabolism in both comparisons (**Figures 3F, G**).

Specifically, three bile acids were decreased and four were increased in the SF group compared with the PSO mice (**Figure 3H**). Correlation analysis indicated that several bile acids were positively associated with *Lactobacillus* abundance, whereas one showed a negative correlation (**Figures 3I, J**). These findings suggest that fecal metabolic changes in dampness-heat psoriasis primarily involve bile acid dysregulation linked to reduced *Lactobacillus*.

### 3.4 Serum metabolic characteristics of psoriasis mice with endogenous dampness-heat syndrome

**Figure 4:**
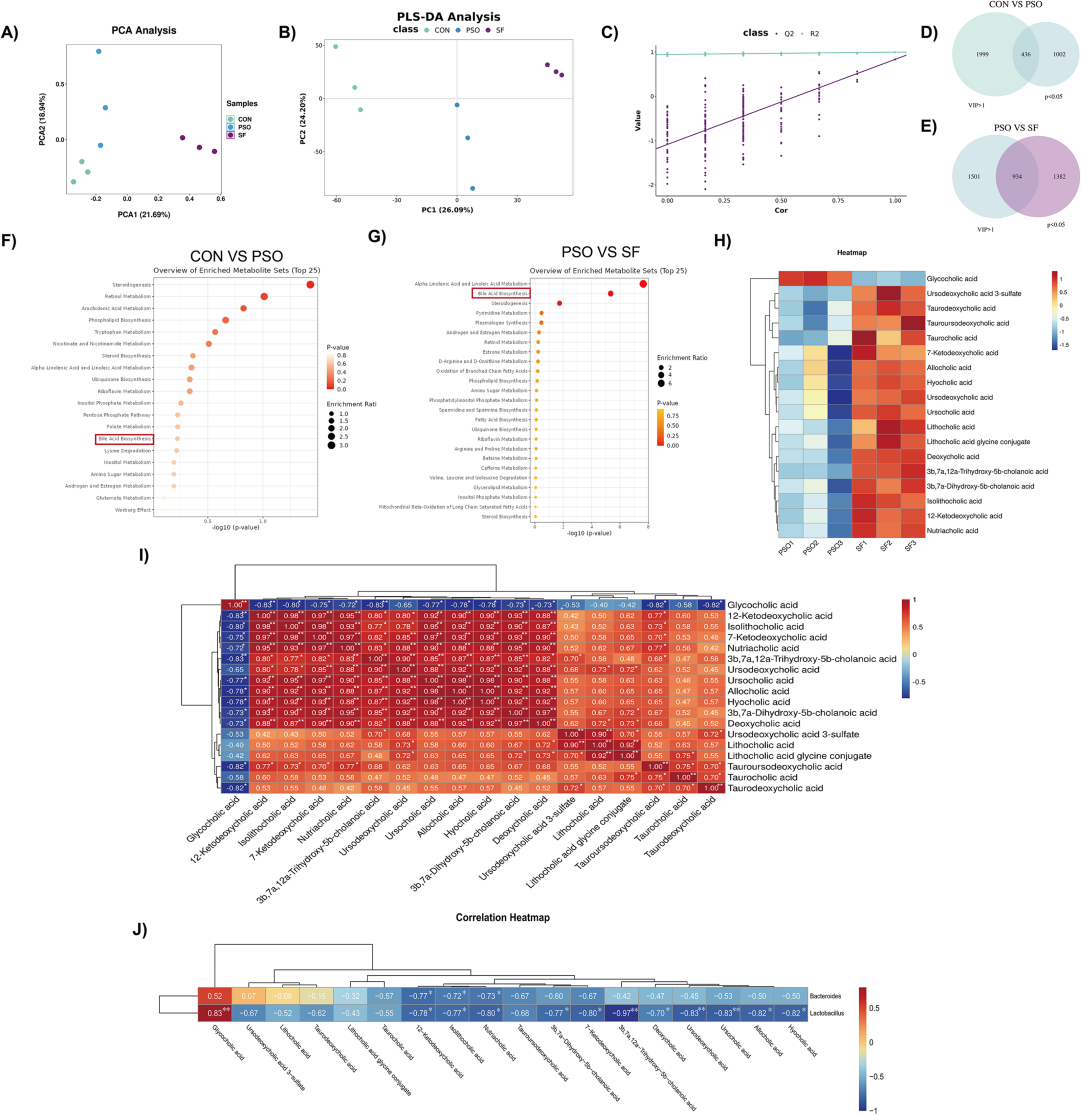
Differences of serum metabolites among the three groups (n=3). **(A)** PCA plot shows the overall distribution and clustering of serum metabolite profiles among the CON, PSO and SF groups. **(B)** PLS-DA score plot illustrates group separation based on serum metabolomic profiles. **(C)** Permutation test of the PLS-DA model evaluates model reliability. **(D)** Using VIP > 1 and *p < 0.05* as selection criteria, the Venn diagram illustrates the number of differentially serum metabolites between the CON (green) and PSO (blue) groups. **(E)** Using VIP > 1 and *p < 0.05* as selection criteria, the Venn diagram illustrates the number of differentially serum metabolites between the PSO (blue) and SF (purple). **(F)** Pathway enrichment plot of differentially serum metabolites between the CON and PSO groups based on the KEGG database. **(G)** Pathway enrichment plot of differentially serum metabolites between the PSO and SF groups based on the KEGG database. **(H)** Heat maps showing the difference of bile acids in each serum sample between the PSO group and the SF group. **(I)** Correlation heat map showed the correlation between differential bile acids. **(J)** Correlation analysis between gut microbiota and bile acids. *p < 0.05, **p < 0.01.

The serum metabolic characteristics of mice with endogenous dampness-heat psoriasis were also analyzed. PCA and PLS-DA analyses indicated distinct serum metabolic characteristics among the groups (**Figures 4A, B**), and PLS-DA permutation test evaluating model reliability and overfitting (**Figure 4C**). Differential serum metabolites between the CON and PSO groups and the PSO and SF groups were determined using univariate (*p < 0.05*) and multivariate statistics (VIP > 1). A total of 436 metabolites differed between CON and PSO groups, and 934 between PSO and SF groups (**Figures 4D, E**). Consistent with the fecal metabolomics findings, pathway analysis highlighted bile acid metabolism as a major altered pathway in both comparisons (**Figures 4F, G**).

Seventeen bile acids were elevated and one decreased in the SF mice compared with the PSO mice (**Figure 4H**). Most were secondary bile acids, including taurocholic acid, ursodeoxycholic acid, cholic acid, ketodeoxycholic acid, and chenodeoxycholic acid. In addition, multiple deoxycholic acids (DCA) accumulated in the serum. Correlation analysis further found that the bile acids of the same class were found to have a clear correlation (**Figure 4I**). Among the eighteen differentially abundant bile acids identified in serum samples (**Figure 4H**), eleven exhibited significant negative correlations with the *Lactobacillus*, while one demonstrated a positive correlation. Additionally, three types of bile acids were negatively correlated with *Bacteroides* (**Figure 4J**). These data suggest that mice with dampness-heat psoriasis accumulate secondary bile acids, particularly DCA, likely linked to reduced *Lactobacillus*.

### 3.5 Bile acid pathway alterations in endogenous dampness-heat psoriasis

**Figure 5:**
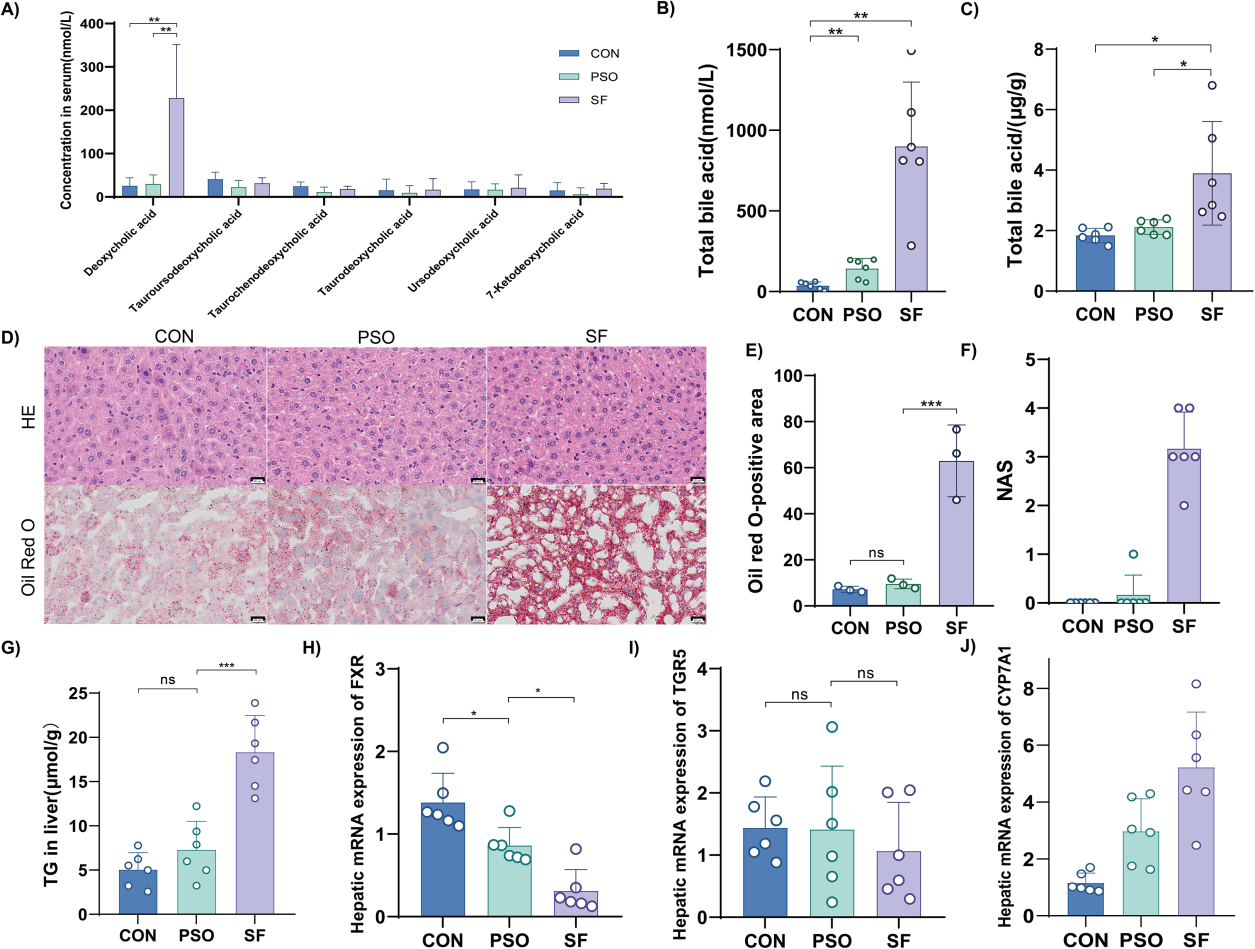
Analysis of bile acids and liver lipids in the 3 groups. **(A)** Serum concentration of selected bile acids (n=6). **(B)** Total serum bile acid (n=6). **(C)** Total liver bile acid (n=6). **(D)** Representative H&E and Oil Red O staining of liver tissue. Scale bar = 20 μm. **(E)** Quantification of Oil Red O–positive area (n=3). **(F)** NAS evaluation. **(G)** Level of TG in liver. **(H-J)** Hepatic mRNA expression of FXR, TGR5 and CYP7A1. *p<0.05, **p<0.01, ***p<0.001.

Targeted metabolomics was conducted to validate bile acid changes, especially DCA. The results were not entirely consistent with those of non-targeted metabolomic analysis. No significant differences were observed for tauroursodeoxycholic acid, taurochenodeoxycholic acid, taurodeoxycholic acid, ursodeoxycholic acid, and 7-ketodeoxycholic acid among the groups. In contrast, DCA was significantly increased in the SF group (*p < 0.05*). Since DCA promotes the accumulation of bile acids and sterols in the body and has pro-inflammatory effects, we measured total bile acids in serum and liver. Consistent with its pro-inflammatory role, total bile acids were indeed abnormally elevated in the serum and liver of the SF group. Oil Red O staining, NAS evaluation and TG assay revealed increased lipid accumulation in the SF group (**Figures 5D–G**). FXR and TGR5 are the primary receptors mediating bile acid involvement in glucose and lipid metabolism. We found that FXR expression was decreased in the SF group, while expression of the downstream bile acid synthase CYP7A1 was increased. No significant difference was observed in TGR5 expression (**Figures 5H–J**). These findings indicate that exacerbated psoriasis symptoms in dampness-heat syndrome mice may be associated with abnormal DCA accumulation and FXR pathway inhibition.

## 4 Discussion

In this study, we propose a mechanistic link between stimulating food–induced endogenous dampness-heat syndrome and psoriasis aggravation. This link is mediated by coordinated alterations in the gut microbiota–bile acid–host metabolic axis. Using a diet-induced endogenous dampness-heat psoriasis model (Yihan et al., 2021; Zeng et al., 2023), we demonstrate that dietary stimulation exacerbates psoriatic inflammation. This effect is associated with disruption of gut microbial composition and bile acid homeostasis. Notably, we observed a marked reduction in *Lactobacillus* abundance, accompanied by accumulation of DCA and dysregulation of hepatic bile acid and lipid metabolism.

At the microbial level, *Lactobacillus* is a beneficial commensal that maintains intestinal barrier integrity and immune homeostasis (Xiao et al., 2021; Shah et al., 2024). Its depletion has been repeatedly observed in dampness-heat syndrome and psoriasis, and is closely associated with enhanced Th17-mediated inflammatory responses (Chen et al., 2021; Chen et al., 2024; Yu et al., 2025; Zhan et al., 2025). In line with previous clinical and experimental studies (Atabati et al., 2020), we observed a significant reduction of *Lactobacillus* in endogenous dampness-heat psoriasis mice, suggesting that loss of this protective genus may predispose the host to inflammatory and metabolic disturbances.

Metabolomic analysis identified bile acid dysregulation as a central metabolic feature of endogenous dampness-heat psoriasis, with DCA emerging as a key metabolite. DCA is a secondary bile acid generated by specific gut bacteria, including Clostridium species, and has been implicated in intestinal inflammation, tumorigenesis, and liver metabolic disorders (Forsgård et al., 2014; Shibuya et al., 2021; Yang et al., 2021; Cong et al., 2024; Liu et al., 2025). Importantly, DCA has been shown to directly inhibit *Lactobacillus* growth, thereby exacerbating intestinal inflammation (Xu et al., 2021; Jin et al., 2022). Our correlation analyses support this interaction, indicating an association between elevated DCA and reduced *Lactobacillus*, thereby perpetuating a cycle of dysbiosis and inflammation.

At the molecular level, excessive DCA accumulation may exert pathogenic effects through suppression of FXR. FXR is a receptor that plays a pivotal role in regulating bile acid synthesis, lipid metabolism, and inflammatory responses (Fu et al., 2019; Jin et al., 2022). In our model, decreased hepatic FXR expression and upregulated CYP7A1 indicate impaired FXR signaling and loss of negative feedback control (Li et al., 2025). This DCA–FXR–CYP7A1 axis provides a plausible molecular mechanism linking gut microbial alterations to hepatic metabolic dysfunction (Jiao et al., 2015).

Previous studies in psoriasis have reported lipid metabolism abnormalities and hepatic steatosis, while our findings further highlight the underlying causes of total bile acid and lipid accumulation (Hao et al., 2022; Sonomoto et al., 2023; Zhao et al., 2023). This variation may be related to the animal model (K14-VEGF mice or IMQ-induced mice), the intervention method (high-fat diet, microbiota transplantation, or stimulating food), the observation period (long-term observation or acute induction), and the sample type (feces or liver). Those studies found that significant steatosis and dysbiotic metabolic disorders emerged during the progression of psoriasis. Our findings extend these observations by identifying excessive DCA accumulation and FXR inhibition as potential upstream drivers.

Taken together, this study provides a holistic mechanistic framework in which stimulating food–induced endogenous dampness-heat syndrome promotes psoriasis aggravation through disruption of the gut microbiota–bile acid–hepatic metabolic axis. These findings highlight a mechanistic link between dampness-heat syndrome and psoriasis aggravation, bridging TCM concepts with modern microbial-metabolic pathways.

Nevertheless, several limitations should be acknowledged. First, this study was based on a murine model, and the relevance of these findings to human psoriasis requires further validation. Second, causal relationships between DCA accumulation, *Lactobacillus* reduction, and FXR expression remain to be directly established. Finally, although our analysis focused on bile acids, other microbial metabolites may also contribute to the observed effects. Future studies integrating gnotobiotic models, microbiota transplantation, and targeted bile acid interventions will be essential for clarifying causal mechanisms and exploring therapeutic strategies.

## 5 Conclusions

In summary, this study employed 16S rDNA sequencing together with non-targeted and targeted metabolomics to characterize gut microbiota and metabolic alterations in mice with endogenous dampness-heat psoriasis. The results demonstrated a marked reduction in *Lactobacillus* and an accumulation of DCA, accompanied by aggravated psoriatic lesions and hepatic lipid deposition. By bridging traditional TCM concepts with contemporary molecular and microbiome research, our findings may help guide future approaches for syndrome differentiation and support the development of microbiota- or metabolite-targeted therapeutic strategies for psoriasis.

## Data Availability

The data presented in the study are deposited in the Nutstore repository, with links attached in **Supplementary Data 2**.
