# Supplementary material for: Diet-induced dampness-heat psoriasis is characterized by reduced Lactobacillus and accumulation of deoxycholic acid
Source: Front Cell Infect Microbiol. 2026 Mar 2;16:1704547. doi: 10.3389/fcimb.2026.1704547 (PMC12989491; doi:10.3389/fcimb.2026.1704547)
Supplement: Supplementary file 1 [file Table1.docx]

**Supplementary Table S1.**

**Detailed calculation of caloric contribution and percentage of recommended daily allowance (%RDA) for each dietary component**

| **Ingredient** | **Amount (g/kgdiet)** | **Energy factor**  **(kcal/g)** | **Energy contribution (kcal/kg)** | **Reference RDA**  **(g/kg diet)*** | **%RDA** | **Calculation notes** |
| --- | --- | --- | --- | --- | --- | --- |
| Casein (30 mesh) | 200 | 4 | 200×4=800 | 140 | 143 | Protein source |
| L-Cystine | 3 | 4 | 3×4=12 | 3 | 100 | Sulfur amino acid |
| Corn starch | 397 | 4 | 397×4=1588 | 397 | 100 | Carbohydrate |
| Maltodextrin 10 | 132 | 4 | 132×4=528 | 132 | 100 | Carbohydrate |
| Sucrose | 100 | 4 | 100×4=400 | 100 | 100 | Carbohydrate |
| Cellulose | 50 | 0 | 0 | N/A | N/A | Non-digestible fiber |
| Soybean oil (no additives) | 70 | 9 | 0 | 630 | 100 | Lipid source |
| t-Butylhydroquinone | 0.014 | 0 | 0 | N/A | N/A | Antioxidant |
| Mineral Mix S10022M | 35 | 0 | 0 | 35 | 100 | Micronutrients |
| Vitamin Mix V10037 | 10 | 0 | 0 | 10 | 100 | Vitamins |
| Choline bitartrate | 2.5 | 0 | 0 | 2.5 | 100 | Essential nutrient |
| Total | 1,000 | - | 3958 | - | - | - |

* The Recommended Daily Allowance (RDA) values were based on the AIN-93M purified rodent diet formulation, which is widely accepted as the standard maintenance diet for adult laboratory mice.

**Energy conversion factors were calculated as follows:**

Protein: 4 kcal/g

Carbohydrate: 4 kcal/g

Fat: 9 kcal/g

%RDA was calculated as:(Actual amount in diet / Recommended amount) × 100

Casein content exceeded 100% RDA to ensure adequate protein supply and nitrogen balance, which is consistent with standard purified rodent diets.

Cellulose and t-butylhydroquinone were included as non-nutritive components and therefore were not subject to %RDA calculations. Choline bitartrate was added at the AIN-93M recommended level and meets 100% of the recommended allowance.
