# Supplementary material for: Diet-induced dampness-heat psoriasis is characterized by reduced Lactobacillus and accumulation of deoxycholic acid
Source: Front Cell Infect Microbiol. 2026 Mar 2;16:1704547. doi: 10.3389/fcimb.2026.1704547 (PMC12989491; doi:10.3389/fcimb.2026.1704547)
Supplement: Supplementary file 2 [file Table2.docx]

16S: <https://www.jianguoyun.com/p/Dd2KtqcQ0-WFDhiwhZoGIAA>

Metabolomics: <https://www.jianguoyun.com/p/DdBpdzoQ0-WFDhizhZoGIAA>
